# Association between Campus Walkability and Affective Walking Experience, and the Mediating Role of Walking Attitude

**DOI:** 10.3390/ijerph192114519

**Published:** 2022-11-05

**Authors:** Bojing Liao, Yifan Xu, Xiang Li, Ji Li

**Affiliations:** 1 Institute of Creativity and Innovation, Xiamen University, Xiamen 361005, China; 2 Economics and Management School, Wuhan University, Wuhan 430072, China; 3 School of Architecture and Civil Engineering, Xiamen University, Xiamen 361005, China; 4 School of Architecture, Southwest Jiaotong University, Chengdu 610031, China

**Keywords:** campus walkability, affective walking experience, walking attitude, structural equation model

## Abstract

The walkability of a neighborhood is important for alleviating transport problems and improving the social and physical wellbeing of residents. However, it is unclear to what extent high walkability contributes to positive attitudes about walking and walking experiences on university campuses. In addition, little is known about the extent and mechanism by which walking attitude influences the affective walking experiences of students. Therefore, this study aimed to analyze the relationship between campus walkability and students’ affective walking experience, as well as to explain the role of walking attitude as a mediator of this relationship. To address these issues, data were collected via questionnaires at a Chinese university and analyzed by using the structural equation model. After controlling for personal characteristics, the results indicated that campus walkability had a positive direct and indirect (through walking attitude) association with affective walking experiences. Our findings have proved that walkable campuses are important because they promote positive walking attitudes and walking emotions, which are beneficial to students’ mental health and subjective wellbeing.

## 1. Introduction

As an important environmental characteristic affecting the built environment, walkability, a measure of the friendliness of a built environment related to physical activity and active mobility [1,2], has been extensively utilized in the fields of public health, transportation, and urban design [3,4,5]. Particularly, walkability is the quality of a neighborhood that supports and encourages people to walk to their destinations in a safe, convenient, and timely fashion [1,6,7]. Walkability thus is often assessed with environmental criteria such as street design, destination accessibility, and safety [8,9,10,11]. A walkable neighborhood environment promotes physical activities that boost social cohesion and economic prosperity through energy savings, cost reductions, and health benefits [12,13]. As a result, major organizations and countries, such as the World Health Organization and the United Nations Centre for Regional Development, consider walkability as one of the key activities to cope with transportation challenges and enhance the social and physical well-being of neighborhood residents [1,14,15].

Walkability is also an important consideration among college students because walking is the main mode of their daily transportation. Walking can also help students meet their daily recommendation of 30 min or more of moderate physical activity during the school or workday, as well as maintain a healthy body weight [8,11,16,17,18,19]. Compared to a neighborhood environment, a campus environment has a distinct boundary, secured entrances, and services for certain individuals (students, faculty, and staff) [11]. Campus environments provide students with amenities that encourage an active lifestyle since they often contain a large area and integrated infrastructure, such as sidewalks, bikeways, and streets [19,20]. Campus environments thus facilitate the creation of lifelong health habits, such as physical activity participation, for many young adults [21,22,23]. Walkability on campus is often determined by both the built environment and students’ perceptions of the environment [11,18,21]. A wide diversity of empirical studies provide evidence on how campus walkability influences physical activity levels among students at different campuses [5,16,18,19,24,25,26,27]. For example, Sisson et al. (2008) revealed that students living on a campus with major academic areas, including many on-campus destinations and restricted parking lots, had a higher walking intensity than those living on a campus with limited access to destinations and streets access to motorized traffic [8]. Likewise, Molina-Garcia et al. (2010) and Vale et al. (2018) have addressed the association between campus walkability and travel-mode commuting, indicating that the spatial location of destinations’ walkability influences the extent of access to various destinations [28,29].

Aside from physical activity, previous studies also support the association between neighborhood walkability characteristics (e.g., land use mix diversity, connectivity, and aesthetics) and walking experience (self-reported affective experience). Because walking promotes not only social capital and a sense of community, but also social interaction and an enhanced sense of safety, these all contribute to a neighborhood’s livability and inhabitants’ mental health [30,31,32,33,34]. On the level of individuals, affective experience is a part of the conceptualization of well-being that is interpreted through the lens of an individual’s perceptions and experiences, which is typically divided into two components: hedonic (satisfaction and positive emotions), and eudemonic (purpose, meaning, or self-actualization) [35,36]. Existing research has found that the characteristics of a neighborhood can contribute to its walkability and have a positive correlation with the affective walking experience [34,35,37,38,39,40,41,42,43,44,45,46,47,48]. These neighborhood walkability characteristics include traffic speed and density, pedestrian safety, street connectivity, residential density, land use mix, and greenery [34,35,38,43]. However, less attention has been paid to the relationship between campus walkability and students’ affective walking experiences. Although a few researchers have examined the influence of campus walkability on affective walking experience, their results are inconsistent. For instance, Lee et al. (2020) have reported that campus connectivity, good-quality sidewalks, and green space were positively associated with the walking experience on a Korean urban campus [49]. In contrast, Liow et al. (2016) have pointed out that a walkable campus environment does not give students a positive walking experience at Universiti Malaya [50]. These arguably contradictory findings are not surprising given the systemic disparities that exist across regions [51]. Nevertheless, more evidence is needed to explain the relationship between campus walkability and students’ affective walking experience.

Furthermore, some prior research has shown the importance of walking attitude in influencing both walkability and affective walking experience [52,53,54,55]. In the most common theories, walking attitude has an influence on the individuals’ perception of walkability, which in turn further affects their walking behaviors and walking experiences [1,36,55,56]. As Y. Yong et al. (2011) have indicated, an individual’s attitude towards walking is influenced by past experiences and characteristics of the built environment [57]. However, recent studies on travel behaviors have begun to examine the reversed causality, that is, that behaviors causally impact attitude [58]. Lin et al. (2017) have provided empirical evidence on the influence of the perception of the built environment on travel behaviors via the attitude of respondents [59]. To that end, it makes sense that a person would live in a highly walkable area for reasons other than neighborhood walkability. Therefore, this person will begin to walk more and develop a positive attitude towards walking behaviors [60]. In other words, it is assumed that varied walking attitudes are influenced by walking behaviors, which are influenced by walkability, and then walkability affects walking attitude. Logically, a positive walking attitude is more likely to result in a positive walking emotion. Consequently, walkability can influence the affective walking experience via the walking attitude. However, the particular nature of the function of walking attitude in the relationship between campus walkability and walking experience is little explored in campus environments.

Although empirical studies have provided insights into the association between walkability and affective walking experience, it is still not clear to what extent high walkable areas contribute to positive walking attitudes and affective walking experiences in campus environments. In addition, little is known about the extent to which, and the mechanism by which, walking attitude contributes to students’ affective walking experiences. Accordingly, the purpose of this research is to investigate the relationship between campus walkability and students’ affective walking experience and to explicate the mediating role of walking attitude in this relationship. To address these issues, data were collected through a survey on the campus of a Chinses university, and the structural equation model was used to analyze data. As it has been demonstrated that personal factors (such as age, length of residence, gender, and level of education) influence walkability and walking attitude [44], the analysis should be corrected for these variables.

**Figure 1:**
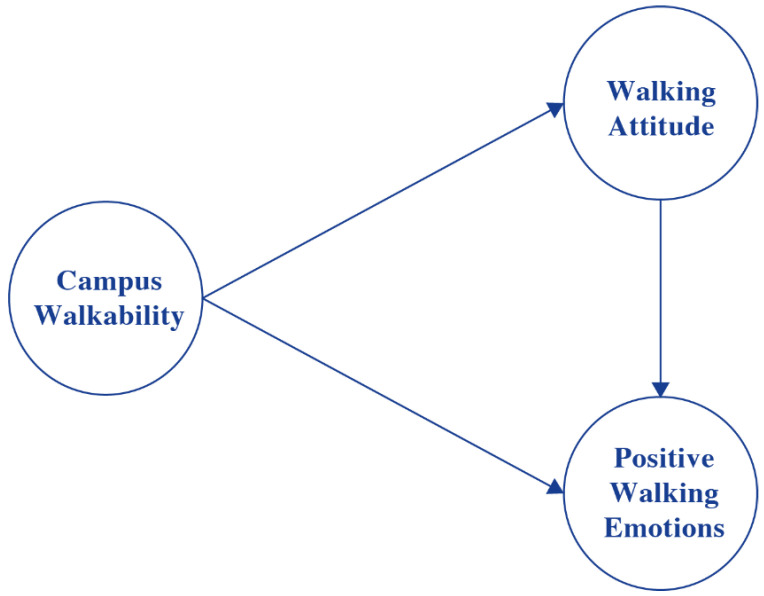
Conceptual model of the relationships between campus walkability, walking attitude, and affective walking experience (positive walking emotions).

Figure 1 illustrates the conceptual model used to investigate the possible relationships in a structural model. In the model, campus walkability has a direct association with walking attitude and affective walking experience (positive walking emotions). The indirect relationships run through the relationships between campus walkability and affective walking experience (positive walking emotions) via the walking attitude. The hypotheses we test can be formulated as follows:

 **Hypothesis 1 (H1).**  *Campus walkability has a direct relationship with walking attitudes, i.e., the higher the walkability the more positive the walking attitude towards walking.*

 **Hypothesis 2 (H2).**  *Campus walkability has a direct relationship with affective walking experiences (positive walking emotions), i.e., the higher the walkability the more positive the walking experience.*

 **Hypothesis 3 (H3).**  *Walking attitudes play an important mediating role in the relationship between campus walkability and affective walking experiences, i.e., the higher the walkability the more positive the walking attitude, and thus the more positive the walking experience.*

Understanding the relationship between campus walkability, walking attitude, and affective walking experience will help researchers and planners gain a better understanding of how to promote students’ feelings about a campus environment. This can contribute to the stability of the campus and the mental well-being of students. Due to the use of cross-sectional data, our analysis does not allow us to identify causality.

The remaining parts of the article are structured as follows. The next section describes the research region, measurement instrument, and data collection for estimating and testing the structural equation model. The method of analysis and results are described in Section 3. In Section 4, noteworthy findings based on the results are discussed. The last section highlights the major conclusion and discusses ways for further study.

## 2. Materials and Methods

### 2.1. Study Site and Survey Instrument

**Figure 2:**
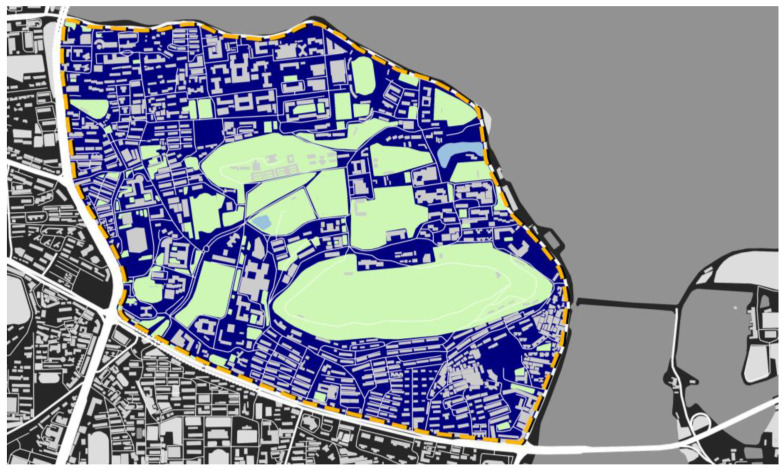
Study site and boundary of the campus, Wuhan University, Wuhan, China.

Our study site was the main campus of Wuhan University in Wuhan, China. The campus is located in the Wuchang District, which has the largest population density in Wuhan City. The campus covers over 320 hectares and has a total building area of over 2.8 billion square meters. Wuhan University has more than 58,000 students enrolled in 34 schools and colleges, and the campus is bisected by Loujia Hill—which makes it one of the most attractive campuses in China. The main campus is surrounded by East Lake, a large green space, and numerous buildings, as shown in Figure 2. As the scenic spot, a range of facilities and services are dispersed around the campus.

A questionnaire was designed to collect data on campus walkability, walking attitude, and affective walking experience in order to investigate the relationships between key concepts in the study site. Campus walkability is measured using the Chinese version of the abbreviated Neighborhood Environment Walkability Survey (NEWS-A) [61]. This survey included five multi-item subscales (This research excludes the perceived residential density because it is based on the type of residences. All participants in this study are students who reside in four-person student apartments.) (land use mix-diversity; access to services; street connectivity; infrastructure and safety for walking; aesthetics; traffic safety; and safety from crime) and four single items (access to parking; hilly streets; physical/natural obstacles; and presence of dead-end streets) [61]. Except for the land use mix-diversity subscale, the other four subscales were rated on a scale of strongly disagree (1) to strongly agree (5). The land use mix diversity is measured by the walking distance from the apartment to different kinds of retailers and facilities, with answers ranging from 1 to 5 min to over 30 min [61,62,63,64]. Higher scores on this scale imply average proximity that is closer. To assess walking attitude, we used the scale of the following items from Cao et al. (2006): “I like walking” and “If possible, I [would] rather walk than drive” [55], with scores ranging from strongly dislike (1) to strongly like (5). For the affective walking experience, empirical studies have found that four dimensions of emotions are associated with perceived walkability (walking through experience): happiness, comfort, annoyance, and security [41,43,65,66]. Because the Chinese version of the NEWS-A is the perceived measurement of walkability, we measured the affective walking experience using these four dimensions of emotions. The questions were structured as statements, as in “I felt happy/comfortable/annoyed/secure,” and the responder rated each item on a 5-point Likert scale ranging from strongly disagree (1) to strongly agree (5). Participants were also asked to fill in their gender, education level, length of campus residence, and cultural background (native or not) in the questionnaire. To ensure that the coherence latent variables were generated appropriately, we reversed coding for some measurement items (all scores from low to high indicated negative situation to positive situation).

### 2.2. Data Collection and Sample

The data for this research were collected during April and May of 2021. All respondents were students, enrolled and residing at Wuhan University, who responded to the questionnaire using a nationwide online investigation platform. Respondents are equally recruited from six dormitory areas of the main campus, including the Hubing Area, the Fengyuan Area, the Guiyuan Area, the Meiyuan Area, the Yingyuan Area, and the Songyuan Area. In total, 518 students completed the online questionnaire. To ensure suitable data quality, respondents who submitted the same answers to each question or completed the NEWS-A section in less than 180 s were eliminated. After data filtering, 334 students remained in the sample.

Table 1 shows the descriptive analysis of the sample characteristics. In Table 1, the share of males is 45.2 percent, and the share of undergraduate students is 97 percent in the sample. Regarding the length of campus residence, 45.2 percent of students live on campus for less than one year, 5.7 percent live on campus for more than two years, and around 50 percent live on campus for between one and two years. In addition, 92.5 percent of students are native students who were born and raised in the city of Wuhan.

### 2.3. Data Analysis

A structural equation model (SEM) is estimated to test and analyze the relationships in the conceptual model (Figure 1). The strength of SEM is that several distinct relations between various independent and dependent variables can well be estimated simultaneously, and both latent variables and observed variables can be integrated [67]. In SEM, a measurement model defines how indicators are associated with latent variables, and a structural model specifies how endogenous and exogenous variables are related. To estimate the model, the maximum likelihood method was used in the R package ‘lavaan’ [68]. To obtain the parsimonious model, a backward stepwise process was utilized to eliminate insignificant correlations between personal characteristics and endogenous variables [67]. As a result, a few characteristics, including gender, were removed from the final model since they were not significantly associated with any of the endogenous variables.

To test the validity and reliability, we conducted the composite reliability (CR) and average variance extracted (AVE) for the measurement items of each latent variable. A CR of 0.70 or more and an AVE of 0.50 or more were recommended [69,70]. For the SEM model as a whole, multiple measurements of the goodness of fit of the estimated model were used. Among them are the Chi-square statistic divided by the degrees of freedom (Chi-square/df), the Goodness-of-Fit Index (GFI), the Normed Fit Index (NFI), the Root Mean Square Error of Approximation (RMSEA), and the Standardized Root Mean Square Residual (SRMR). Acceptable model fit values are >0.90 for NFI and GFI, <5 for Chi-square/df, and <0.08 for both RMSEA and SRMR [71,72,73].

## 3. Results

This section presents the results for the standardized estimates of measurement items of latent variables and the estimation results of the SEM model. The coefficients of the relationships between the main latent variables and measurement items, as well as the validation measures of composite reliability and average variance extracted for the latent variables in the final model, are shown in Table 2. Also, Table 2 indicates that the CR and AVE values of the main latent variables are greater than the recommended levels.

In Table 3, the results of the structural model are presented, including the goodness-of-fit statistics of the model. For the goodness-of-fit values of the model, the value of Chi-square/df is 2.93, the values of GFI and NFI are 0.905 and 0.925, and the values of RMSEA and SRMR are 0.066 and 0.078, respectively. The results suggest that the model has an adequate fit for the data. The R^2^ values for walking attitude and affective walking experience are 0.185 and 0.612, respectively, and their related adjusted R^2^ values are 0.185 and 0.418, as shown in Table 3.

Preliminary bivariate analyses showed significant positive correlations between campus walkability and walking attitude, between walking attitude and affective walking experience (positive walking emotions), and between campus walkability and affective walking experience. When the control variables are included, there is no significant relationship between personal characteristics and campus walkability, as well as personal characteristics and affective walking experience. Only walking attitude is negatively related to education level (undergraduate students) and geographic background (native students). Additionally, the length of campus residence has a positive relationship with walking attitude.

In this research, campus walkability has a positive relationship with both walking attitude and affective walking experience (positive walking emotions). The results further indicate that campus walkability has a direct relationship with affective walking experience and an indirect relationship with affective walking experience through walking attitude. It indicates that campus walkability influences walking attitude, which in turn influences affective walking experiences. The direct relationship between campus walkability and affective walking experience is stronger than the indirect relationship through walking attitude in Table 3. These findings suggest that an increase in campus walkability is associated with an increase in students’ positive walking experiences, both directly, and indirectly through walking attitude, in the case of campus walkability.

## 4. Discussion

**Figure 3:**
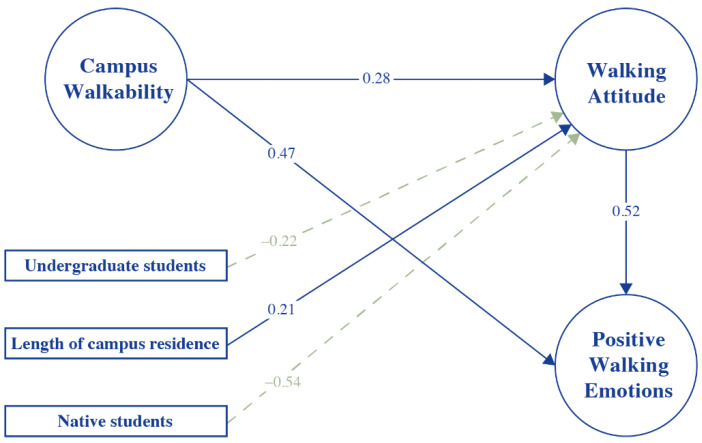
Standardized direct path coefficients SEM.

By coupling student surveys with the SEM results, this study can test and explain the hypotheses we presented in Section 2, as shown in Figure 3.

In terms of the H1, the SEM results suggest a positive relationship between campus walkability (perceived) and walking attitude (β = 0.28). This finding supports the hypothesis that the higher the walkability on campus, the more positive the students’ walking attitude towards walking. It is partly consistent with Molin et al. (2016) and Faber et al. (2021), who found that built environment factors impacted residents’ travel attitudes [58,60]. In particular, it somewhat reversed the extent of common walkability theories. According to common walkability theories, walking attitude influences perceived walkability, which in turn influences walking behaviors in a neighborhood. However, it is reasonable that a person may reside in a very walkable place for reasons other than perceived walkability, especially on a university campus. The study site—Wuhan University—is one of the most beautiful campuses in China, with a high residential density, connectivity, and greenness, making the campus highly walkable [74]. As many Chinese Universities do, Wuhan University also required its students to live on the campus. A student who lives in this gated university can walk more and enjoy the highly walkable environment more. Consequently, depending on his or her walking behavior, he or she is developing a more positive attitude towards walking [58].

Regarding the H2, our results indicate a significant correlation between walkability and affective walking experience (positive walking emotions, β = 0.47). It supported the empirical findings of Ettema et al. (2015), Weijs-Perr’ee et al. (2020), and Liao et al. (2022), wherein it has been revealed that a sense of happiness, a sense of security, and a sense of comfort are associated with walkability. This implies that campus environments influence the emotions of students, coloring their individual perceptions of walkability on campuses. As a result, the higher the campus walkability, the more positive students’ walking experiences.

For the H3, Figure 3 illustrates that campus walkability is related to walking attitude and to affective walking experiences. The walking attitude plays a slight mediating role (β = 0.10) in the relationship between the campus walkability and affective walking experience. Hence, it is partly supporting Hypothesis 3. Moreover, the walking attitude is strongly associated with the affective walking experience (β = 0.52), which is consistent with many empirical studies indicating that an individual’s attitude influences their emotions [32,41,58,65].

About the personal characteristics added as control variables, several significant relationships have been observed, which are consistent with empirical findings. First, education level is negatively associated with the walking attitude, suggesting that undergraduates have a low preference for walking. Following the findings of Ding et al. (2017), Chinese young adults are less likely to consider walking and cycling as their main mode of transportation [75]. There is another negative relation between cultural background and walking attitude, which means that native students are less likely to prefer walking than outlanders on a campus. In addition to negative relationships, the length of residence on campus has a positive relationship with walking attitude, indicating that familiarity with the environment impacts the attitude of students on campus. It is in line with empirical studies on place attachment, individuals tend to develop an emotional attachment to their living environment and maintain a close relationship with it over time [76].

## 5. Conclusions

The purpose of this study is to provide insights into the relationship between campus walkability and affective walking experiences and the mediating role of walking attitude. In order to examine the direct and indirect association between campus walkability and affective walking experience, we estimated a structural equation model using a data set from Wuhan University (N = 334) by controlling for personal characteristics.

After controlling for personal variables, the results indicated that campus walkability has both a positive direct and indirect (through walking attitude) relationship with affective walking experiences. Our findings suggest that walkable campuses are important because they promote positive walking attitudes and walking emotions, which are beneficial to the mental health and subjective wellbeing of students.

According to the model, campus walkability is an efficient predictor of both walking attitude and affective walking experience; hence, campus administrators should concentrate on increasing (perceived) campus walkability. For campus walkability, proximity to service points, maintained pedestrian pavement, green space, attractive buildings, interesting things to look at, and safety traffic speed are important. These walkability components may promote positive walking attitudes and feelings on campus. As our study measured only perceptions of campus walkability, more research is needed on the relationship between objective campus walkability and affective walking experience.

In addition, we found no relationship between affective walking experience and personal characteristics, and the few personal characteristics we employed were control variables in our study. We did not include associations between personal variables and campus walkability in the model. For future research, it would be interesting to explore how different student groups with diverse personal backgrounds perceive walkability.

The results of this study are based on data collected at Wuhan University, which is a scenic spot and is located in the central area of China. This means that the findings are also applicable to other colleges in China with similar geographic backgrounds, such as Huazhong Normal University, Hunan University, Xiamen University, and others. However, our results may not be generalizable to other non-scenic spot areas in China and other countries. In light of the cultural diversity and breadth of China’s land, it would be fascinating to do similar studies in other regions with various cultural and regionalist backgrounds. Moreover, the conclusions are based on cross-sectional data, therefore causality cannot be demonstrated. To determine if walkability generates positive emotional effects, longitudinal data would need to be collected in future research.

Although further research is needed to complement the findings, this study demonstrated that campus walkability can play an important role in promoting positive walking attitudes and affective walking experiences and therefore contributes to students’ physical and mental wellbeing.

## Figures and Tables

**Table 1:** Descriptive analysis of the sample characteristics.

	Sample (N)	Sample (%)
***Gender***		
Male students	151	45.2%
Female students	183	54.8%
***Education level***		
Undergraduate students	324	97.0%
Graduate students	10	3.0%
***Length of campus residence***		
Less than one year	151	45.2%
1–2 years	164	49.1%
More than 2 years	19	5.7%
***Cultural background***		
Native students	309	92.5%
Non-native students	25	7.5%
***Total***	334	100%

**Table 2:** Standardized Estimates for Measurement Items of Latent Variables.

**Variables**	**Estimate**
***Campus walkability***	
*1. About how long would it take to get from your dormitory to the nearest facilities listed below if you walked to them*	
Classroom buildings	0.75
Library	0.68
Canteen	0.66
Snack bar	0.67
Fruits store	0.74
Delivery points	0.76
Supermarket	0.56
Department store	0.68
Gym	0.75
Outdoor sports space	0.68
Hospital	0.59
School bus stop	0.60
Green space	0.69
** Composite Reliability**	**0.901**
** Average Variance Extracted**	**0.505**
*2. Access to services*	
Classroom buildings are within easy walking distance of my dormitory	0.82
Stores are within easy walking distance of my dormitory	0.71
Commercial areas are easy to arrive by public transportation	0.76
There are many places to go within easy walking distance of my dormitory	0.84
There are many slopes that make the road difficult to walk *	0.57
There are many obstacles that make the road difficult to walk *	0.66
There are many pedestrians on the street at peak hours *	0.67
There are many pedestrians on the street at non-peak hours *	0.66
** Composite Reliability**	**0.893**
** Average Variance Extracted**	**0.513**
*3. Streets on my campus*	
The streets on my campus do not have many, or any, cul-de-sacs	0.39
The distance between intersections on my campus is usually short	0.76
There are many alternative routes for getting from place to place	0.89
** Composite Reliability**	**0.738**
** Average Variance Extracted**	**0.507**
*4. Places for walking*	
There are sidewalks on most of the streets on my campus	0.86
Sidewalks are occupied by parked cars on my campus *	0.21
There is a strip that separates the streets from the sidewalks on my campus	0.76
Streets are well-lit at night	0.62
There are zebra crossings and traffic lights on a busy street	0.67
Walkable indoor spaces (with air-conditioning) are available on my campus	0.79
Streets and sidewalks are usually wet on my campus *	0.81
There are rest facilities, such as benches	0.87
** Composite Reliability**	**0.892**
** Average Variance Extracted**	**0.529**
*5. Campus surroundings*	
There are trees along the streets on my campus	0.43
There are many interesting things to look at while walking on my campus	0.82
There are many green spaces on my campus	0.91
There are attractive buildings on my campus	0.91
Air pollution is usually high on my campus *	0.43
** Composite Reliability**	**0.842**
** Average Variance Extracted**	**0.540**
*6. Safety from traffic*	
There is so much traffic along the nearby street that it makes it difficult to walk *	0.65
The speed of traffic on most nearby streets is usually slow (30 km/h or less)	0.22
Most drivers exceed the posted speed limits while driving on my campus *	0.68
There are many parked cars on nearby streets that which makes it difficult to cross *	0.92
There are many passing cars on nearby streets that it is frightening to cross *	0.94
** Composite Reliability**	**0.833**
** Average Variance Extracted**	**0.533**
***Affective walking experience (positive walking emotions)***	
I felt happy	0.83
I felt annoyed *	0.11
I felt comfortable	0.95
I felt secure	0.59
** Composite Reliability**	**0.768**
** Average Variance Extracted**	**0.514**

* indicates that this measurement item is reverse coded.

**Table 3:** SEM Results—Standardized Coefficients.

To	Walking Attitude		Positive Walking Emotions	
From	Direct	Total	Direct	Total
Campus walkability	0.283	0.283	0.470	0.570
Walking attitude			0.521	0.521
Undergraduate students	−0.221	−0.191		
Length of campus residence	0.206	0.217		
Native students	−0.539	−0.142		
R^2^	0.612		0.185	
Adjusted R^2^	0.418		0.185	
Chi-square/df	2.930			
Goodness-of-fit index	0.905			
Normed fit index	0.925			
RMSEA	0.066			
SRMR	0.078			

Note: SEM = structural equation modeling; RMSEA = root mean square error of approximation; SRMR = standardized root mean square.
